# Bioinformatics combined with quantitative proteomics analyses and identification of potential biomarkers in cholangiocarcinoma

**DOI:** 10.1186/s12935-020-01212-z

**Published:** 2020-04-22

**Authors:** Zijian Da, Long Gao, Gang Su, Jia Yao, Wenkang Fu, Jinduo Zhang, Xu Zhang, Zhaoji Pei, Ping Yue, Bing Bai, Yanyan Lin, Wenbo Meng, Xun Li

**Affiliations:** 1 The First Clinical Medical College, Lanzhou University, Lanzhou, 730000 China; 2 Department of Special Minimally Invasive Surgery, The First Hospital of Lanzhou University, Lanzhou, 730000 China; 3 Division of Scientific Research and Development Planning, The First Hospital of Lanzhou University, Lanzhou, 730000 China; 4 Institute of Genetics, School of Basic Medical Sciences, Lanzhou University, Lanzhou, 730000 China; 5 Gansu Province Institute of Hepatopancreatobiliary, Lanzhou, 730000 China; 6 Gansu Province Key Laboratory Biotherapy and Regenerative Medicine, Lanzhou, 730000 China; 7 The Second Department of General Surgery, The First Hospital of Lanzhou University, Lanzhou, 730000 China

**Keywords:** Cholangiocarcinoma, Bioinformatics, iTRAQ, CASK, Prognosis, Multiomics

## Abstract

**Background:**

Cholangiocarcinoma (CCA) is an invasive malignancy arising from biliary epithelial cells; it is the most common primary tumour of the bile tract and has a poor prognosis. The aim of this study was to screen prognostic biomarkers for CCA by integrated multiomics analysis.

**Methods:**

The GSE32225 dataset was derived from the Gene Expression Omnibus (GEO) database and comprehensively analysed by using R software and The Cancer Genome Atlas (TCGA) database to obtain the differentially expressed RNAs (DERNAs) associated with CCA prognosis. Quantitative isobaric tags for relative and absolute quantification (iTRAQ) proteomics was used to screen differentially expressed proteins (DEPs) between CCA and nontumour tissues. Through integrated analysis of DERNA and DEP data, we obtained candidate proteins APOF, ITGAV and CASK, and immunohistochemistry was used to detect the expression of these proteins in CCA. The relationship between CASK expression and CCA prognosis was further analysed.

**Results:**

Through bioinformatics analysis, 875 DERNAs were identified, of which 10 were associated with the prognosis of the CCA patients. A total of 487 DEPs were obtained by using the iTRAQ technique. Comprehensive analysis of multiomics data showed that CASK, ITGAV and APOF expression at both the mRNA and protein levels were different in CCA compared with nontumour tissues. CASK was found to be expressed in the cytoplasm and nucleus of CCA cells in 38 (45%) of 84 patients with CCA. Our results suggested that patients with positive CASK expression had significantly better overall survival (OS) and recurrence-free survival (RFS) than those with negative CASK expression. Univariate and multivariate analyses demonstrated that negative expression of CASK was a significantly independent risk factor for OS and RFS in CCA patients.

**Conclusions:**

CASK may be a tumour suppressor; its low expression is an independent risk factor for a poor prognosis in CCA patients, and so it could be used as a clinically valuable prognostic marker.

## Background

Cholangiocarcinoma is an invasive malignancy originating from biliary epithelial cells and is the most common primary tumour of the bile tract [1]. Over the past few decades, the incidence of CCA has steadily increased [2]. This trend brings more serious challenges to CCA research. The prognosis of CCA is very poor, and the disease not sensitive to chemoradiotherapy. Surgical treatment is the primary intervention at present [3]. However, patients with CCA often do not manifest obvious symptoms in the early stage, and the vast majority of clinically diagnosed CCA patients have often missed the opportunity for radical surgery [4]. In addition, although the survival rate of patients with radical resection is significantly higher than that of patients without resection, recurrence is still the leading cause of death in such patients, and the 5-year survival rate after surgery is still a depressing figure [5, 6]. Therefore, it is necessary to screen effective prognostic biomarkers to identify high-risk CCA patients and develop appropriate treatment strategies to improve their prognoses.

In recent decades, many biomarkers of CCA have been found through continuous CCA research [7, 8]. Carbohydrate antigen 19-9 (CA19-9) is the most commonly used diagnostic marker in clinical practice, but due to concomitant biliary inflammation and Lewis-gene-negative cases [9], its diagnostic efficacy is limited. Some studies have reported that increased preoperative CA19-9 levels are associated with poor postoperative prognosis in CCA patients [10, 11]. Gardini et al. found that low expression of connective tissue growth factor (CTGF) was an independent risk factor for a poor prognosis in CCA patients [12]. However, these biomarkers are still insufficient to fully understand the molecular mechanisms of CCA and to access CCA prognosis to optimize treatment strategies. We need to use more efficient methods to screen out new, more valuable biomarkers to promote CCA research [13] and develop and improve clinical, precise, individualized treatment schemes.

Isobaric tags for relative and absolute quantification (iTRAQ) technology is one of the most popular methods for differential protein identification; it allows the high-throughput, simultaneous comparison of protein expression among up to 8 samples with high labelling efficiency [14]. However, iTRAQ may yield up to hundreds or thousands of DEPs, and it is difficult to identify which proteins can serve as prognostic biomarkers for CCA. Fortunately, as advances in bioinformatics have continued to be developed, we can make bioinformatics predictions by analysing public CCA data [13]. Integrating the data obtained from iTRAQ and conducting a multiomics analysis can yield proteins that may serve as prognostic markers of CCA for further research. In addition, these prognostic biomarkers may help to further reveal the mechanism of tumourigenesis and development of cancer from a systematic perspective, providing an important basis for the individualized and precise treatments for CCA.

In this study, we obtained 10 mRNAs related to the prognosis of CCA by obtaining CCA data from the Gene Expression Omnibus (GEO) and The Cancer Genome Atlas (TCGA) database and conducting a comprehensive analysis. Through integrated analysis with the proteome data obtained by iTRAQ, we screened three candidate proteins APOF, ITGAV and CASK, and verified their expression with immunohistochemistry. Finally, we identified CASK as a valuable biomarker in CCA patients for predicting the recurrence of CCA and a poor clinical outcome.

## Materials and methods

### Data obtaining and preprocessing

Matrix files from the GSE32225 dataset were obtained from the GEO (https://www.ncbi.nlm.nih.gov/geo/) database. The expression profile of the GSE32225 dataset was recorded in GPL8432 (Illumina HumanRef-8 WG-DASL v3.0) and contained the gene expression data from 149 cancer samples and 6 normal tissues [15]. The gene probes were matched with the gene symbols in the platform annotation file of GPL8432 one by one. When the gene symbols were matched with multiple probes, the gene expressions were averaged.

### Identification of differentially expressed RNAs

R software (version 3.6.0, https://www.r-project.org) was used to process the data. We used the “limma” package to identify DERNAs between CCA tissues and nontumour tissues [16]. Then, the expression values were log2 converted. Adjusted P values (FDR) < 0.05 and | log2FoldChange (FC) | > 1 were established as screening threshold values. Finally, we obtained the DERNAs, which will be used for further analyses.

### Function and pathway analysis

To understand the underlying functions of these DERNAs, we used the Database for Annotation, Visualization and Integrated Discovery (DAVID, https://david.ncifcrf.gov) web tool to perform Gene Ontology (GO) and Kyoto Encyclopedia of Genes and Genomes (KEGG) analyses [17, 18]. The results of enrichment analysis with P values < 0.05 were considered to be statistically significant.

### Survival analysis and validation of expression

To determine whether these DERNAs could be used as biomarkers for the prognosis of CCA, we used Gene Expression Profiling Interactive Analysis (GEPIA, http://gepia.cancer-pku.cm), an analysis tool that provides customized gene expression data based on the TCGA databases, to perform a Kaplan–Meier survival analysis to assess the prognostic value of these DERNAs [19]. The statistically significant threshold was set as P < 0.05. CCA sequencing data from the TCGA database were analysed using GEPIA to verify the differential expression of previously obtained survival-related RNAs.

### Selection of patients and collection of clinical samples

A total of 6 matched primary CCA tissues and paracancerous tissues were collected from patients undergoing surgery in The Department of Special Minimally Invasive Surgery, The First Hospital of Lanzhou University. All of the tissue specimens were diagnosed with primary CCA without radiotherapy or chemotherapy before surgery by two pathologists. These collected samples were used for iTRAQ followed by LC–MS. The basic clinical information of these patients is summarized in Table 1.

**Table 1:** Summary of clinic parameters of CCA patients collected for iTRAQ

Code	Pathology diagnosis	Age	Gender	Grade	Lymph node metastasis
1	Cholangiocarcinoma	51	Female	Well	Positive
2	Cholangiocarcinoma	55	Female	Poorly	Negative
3	Cholangiocarcinoma	51	Male	Poorly	Positive
4	Cholangiocarcinoma	58	Male	Moderately	Positive
5	Cholangiocarcinoma	72	Male	Moderately	Negative
6	Cholangiocarcinoma	75	Male	Moderately	Negative

In addition, 7 cases of paraffin-embedded choledochal cysts and 86 cases of paraffin-embedded CCAs were obtained from patients receiving surgical treatment at The First Hospital of Lanzhou University from 2011 to 2016, and were used for immunohistochemical detection and tissue microarray (TMA) construction, respectively. This experimental scheme was approved by the Ethics Committee for Human Research, Lanzhou University. All tissue samples were obtained with written consent from the participants, and all diagnoses were confirmed by postoperative pathology. The inclusion criteria for CCA patients in this study were as follows: (1) the postoperative pathological diagnosis was primary cholangiocarcinoma; (2) no distant metastasis had occurred; (3) the patient had not undergone chemoradiotherapy before surgery; (4) the patient had undergone radical surgical resection; (5) no life-threatening preoperative and postoperative complications had occurred; and (6) adequate clinical and follow-up data were available. The clinical features of the CCA patients are summarized in Table 2.

**Table 2:** Summary of clinical characteristic of CCA patients collected for TMAs

Characteristic	No. patients (%)
Age	
< 55	34 (39.5)
≥ 55	52 (60.5)
Gender	
Female	43 (50.0)
Male	43 (50.0)
Histologic grade	
Poorly	17 (19.8)
Moderate	64 (74.4)
Well	5 (5.8)
T classification	
T1	6 (7.0)
T2	59 (68.6)
T3	18 (20.9)
T4	3 (3.5)
N classification	
N0	58 (67.4)
N1	28 (32.6)
Vascular invasion	
No	57 (66.3)
Yes	29 (33.7)
Recurrence	
No	31 (36.0)
Yes	55 (64.0)

### Sample preparation and protein quantification

Frozen tissue samples were sectioned after thawing on ice. After adding lysis buffer (containing 0.1 mmol/L phenylmethylsulfonyl fluoride, 2 mol/L thiourea, 4% 3-(3-cholamidopropyl) dimethylammonium propane sulfonate (CHAPS), 7 mol/L urea and 65 mmol/L dithiothreitol) and 4% phenylmethylsulfonyl fluoride (PMSF), the tissue cubes were ground in liquid nitrogen and fully extracted. Then the obtained samples were placed on ice and sonicated for 3 min (0.8-s sonication, followed by a 0.8-s interval) with the power set at 80 W. The samples were thoroughly mixed with prechilled acetone at a prechilled acetone to sample ratio of 5:1 (v/v), and the resulting mixture was precipitated overnight at − 20 °C. Then, the mixture was centrifuged at 12,000×*g* and 4 °C for 10 min. After removing the supernatant, 2 mL prechilled acetone was added to the collected precipitate to shake and mix and the resulting mixture was centrifuged again with the above parameters for 15 min. Then we collected the precipitate and repeated the above steps. After removing the supernatant, the mixture was collected and dried at room temperature. The precipitate was added to 0.5 ml of 1 M triethylammonium bicarbonate buffer (TEAB; Sigma-Aldrich, Australia), and the mixture was thoroughly mixed and dissolved and then centrifuged at room temperature and 12,000×*g* for 15 min. After fully removing the insoluble impurities, the obtained supernatant was transferred to a new 1 mL test tube, and the protein concentration was measured by using the Bradford Protein Assay (Tiangen, Beijing, China).

### Protein digestion and iTRAQ labelling

After protein quantification, prechilled acetone 5 times the volume of each sample was added to 100 µg of protein per group for precipitation, which was then fully precipitated at − 20 °C for 1 h. The resulting mixture was then centrifuged for 10 min at 4 °C and 12,000×*g*, and the precipitate was collected and dried by using a vacuum centrifuge. Then, 50 µL dissolution buffer was added to the precipitate to fully dissolve the protein, reacted with 4 µl reducing reagent for 1 h at 60 °C, and then alkylated with 2 µl cysteine blocking for 10 min at room temperature. Then, 50 µl trypsin (50 ng/µL) was added to the protein sample to fully dissolve it for 12 h at 37 °C. After the tryptic peptide mixtures were dried by vacuum centrifugation, iTRAQ reagent was added to the mixture, which was then labelled for 2 h at room temperature. Then, 100 ml distilled water was added to the samples to stop the reaction, and all the labelled samples were mixed in equal quantities. After the samples were completely dried by vacuum centrifugation, the samples were kept for further isolation and identification. The above iTRAQ labelling steps were performed in accordance with the reagent manufacturer’s instructions (AB SCIEX, Shanghai, China).

### LC–MS/MS analysis

LC–MS/MS was carried out by Sangon Biotech (Shanghai) Co., Ltd. The freeze-dried samples were dissolved in 110 µl Nano-RPLC buffer A (0.1% formic acid and 2% ACN) and peptide separation was conducted by an Agilent 1200 HPLC system on a secondary RP analytical column (Analytical Guard Column 4.6 × 12.5 mm 5-μm; Narrow-Bore 2.1 × 150 mm 5 μm). The sample was then loaded with a flow rate of 0.3 mL/min for 1 h by using a linear binary gradient of 0–80% buffer B (350 mmol/L KCl in solvent A, pH = 2.8). The absorbance of the 6 collected SCX fractions was measured at 210 nm and 280 nm. The peptide mixture was redissolved in Nano-RPLC buffer A, and online Nano-RPLC liquid chromatography was performed using LC-20AD nano-RPLC (AB SCIEX). The analytical column was a C18 reversed-phase chromatographic column (75 μm × 15 cm C18 - 3 μm 120 A, ChromXP Eksigent). Subsequently, the gradient condition with phase B at a flow rate of 300 μL/min (5% phase B for 8 min, 25% phase B for 38 min, 40% phase B for 50 min, 90% phase B for 60 min, 2% phase B for 65 min, and 0 phase B for 70 min) were used to eluted the peptides. Mass spectrometry was performed on a Triple TOF 5600 (AB SCIEX) with 2.5 kV electrospray, and the information independent analysis (IDA) mode was used as the mass spectrometry scanning mode. In IDA mode, a hybrid quadrupole time-of-flight mass spectrometer (QStar hybrid LC/MS/MSQ-TOF, AB SCIEX, China) was operated to switch automatically between MS and MS/MS acquisition. The scanning time of the first level single MS was set as 250 ms, and a maximum of 35 s-level MS with charges of 2 + to 5 + and a single second count of more than 150 were collected under each IDA cycle. Dynamic exclusion was set to 18 s, approximately equal to the chromatographic half-peak width.

### Protein identification and integrated analysis

The LC-MS/MS data were analysed by Sangon Biotech (Shanghai) Co., Ltd using the SEQUEST algorithm. The results were compared with the data in the NCBI Human RefSeq database. A cutoff value > 1.3 and peptides≥ 1 were used as thresholds for protein identification. If the FC value > 1.2 or < 0.87 in the cancer group compared with the nontumour group, the protein was regarded as a differentially expressed protein. A P value < 0.05 FC value for at least one dataset indicated significant differential expression. The validated prognosis-related RNA and the DEP data obtained by iTRAQ were integrated and analysed to obtain the intersection between the two sets of data. The candidate proteins in this intersection were reserved for further analysis.

### Tissue microarray (TMA)

TMAs were constructed by the Pathology Department of the First Hospital of Lanzhou University, for which Shanghai Outdo Biotech Company (Shanghai, China) provided technical support. The TMA contained a total of 122 points, of which 31 included noncarcinoma foci. The paraffin-embedded tissue in each core was obtained from the non-necrotic area of the carcinoma and/or noncarcinoma foci.

### Immunohistochemistry (IHC)

Integrin subunit alpha V (ITGAV) was detected by ITGAV rabbit polyclonal antibody (27,096-1-AP). Apolipoprotein F (APOF) was detected by APOF rabbit polyclonal antibody (16608-1-AP). Both antibodies were obtained from Proteintech (Wuhan, China). Peripheral plasma membrane protein CASK (CASK) was detected by rabbit polyclonal anti-CASK antibody (ab244393), which was purchased from Abcam (Shanghai, China). Goat serum and 3,3’-diaminobenzidine (DAB) substrate were purchased from Fuzhou Maixin Biotechnology Co. (Fuzhou, China). The tissue was embedded and fixed, and then the sections were dewaxed with xylene and diluted and hydrated with ethanol and distilled water, respectively. The sections and TMA were treated with antigenic thermal repair. Sections were incubated at room temperature with 3% hydrogen peroxidase for 10 min to eliminate endogenous peroxidase activity. The section and TMA were sealed with goat serum to prevent nonspecific staining. The sections and TMA were dripped with primary antibodies (ITGAV, 1:200; APOF, 1:50; CASK, 1:20) at 4 °C overnight. The sections and TMA were then dripped with secondary antibody and incubated at room temperature for 30 min. The sections were dripped with fresh DAB substrate and reacted for 5 min, then thoroughly rinsed with tap water, followed by counterstaining with haematoxylin. After dehydration with ethanol and xylene, the tablets were sealed with neutral gum.

### Evaluation of the results by scoring

Immunoreactivity images and their corresponding scores were assessed independently by two pathologists who had no knowledge of the experimental design, and any inconsistent scores were reassessed by both pathologists until a consistent score was obtained. The score for immunoreactivity included staining intensity and positive cell proportions. At 200× magnification, 10 fields were randomly selected for scoring. The staining intensity score was divided into 4 grades from 0 to 3, with 0 indicating invisible positive staining, 1 indicating weak staining, 2 indicating moderate staining and 3 indicating strong staining. The percentage score, which evaluates the estimated percentage of tumour cells with positive staining, was also classified into 4 levels from 0 to 3 (0: non-positive staining; 1: 0–20%; 2: 21–50%; 3: > 51%). The final immunoreactivity score was obtained by multiplying the intensity score by the percentage score. For the statistical analysis, we defined an immunostaining score of 0–2 as negative (0: completely negative; 1–2: weakly positive), while 3–9 was defined as positive (3–5: moderately positive; 6–9: strongly positive).

### Statistical analysis

The Chi squared test, Fisher’s exact test, or *t* test were used to identify the association between CASK expression and the clinicopathological characteristics of patients with CCA. Kaplan–Meier analysis was applied to compare OS and RFS between positive and negative CASK-expression groups, and P values were calculated by the log-rank test to verify the difference in survival curves. Univariate and multivariate Cox regression analyses were applied to explore the potential prognostic risk factors in CCA patients. In the univariate analysis, we used 0.05 as the cutoff P value to select the analysis factors to be included in the multivariate analysis. P < 0.05 was considered statistically significant. All statistical analyses were performed by using SPSS 21.0 (SPSS Inc., Chicago, IL) and the R software package (version 3.6.0, https://www.r-project.org).

## Results

### Identification of DERNAs in CCA

By setting the thresholds for the adjusted P value (FDR) < 0.05 and | log_2_FoldChange (FC) | > 1 for differential expression analysis, we identified a total of 875 significantly DERNAs (372 upregulated and 503 downregulated) in GSE32225 (Additional file 1). The volcano plots (Additional file 2) and heatmap (Additional file 3) of identified DERNAs were up loaded in additional files.

### Functional enrichment analysis

**Fig. 1:**
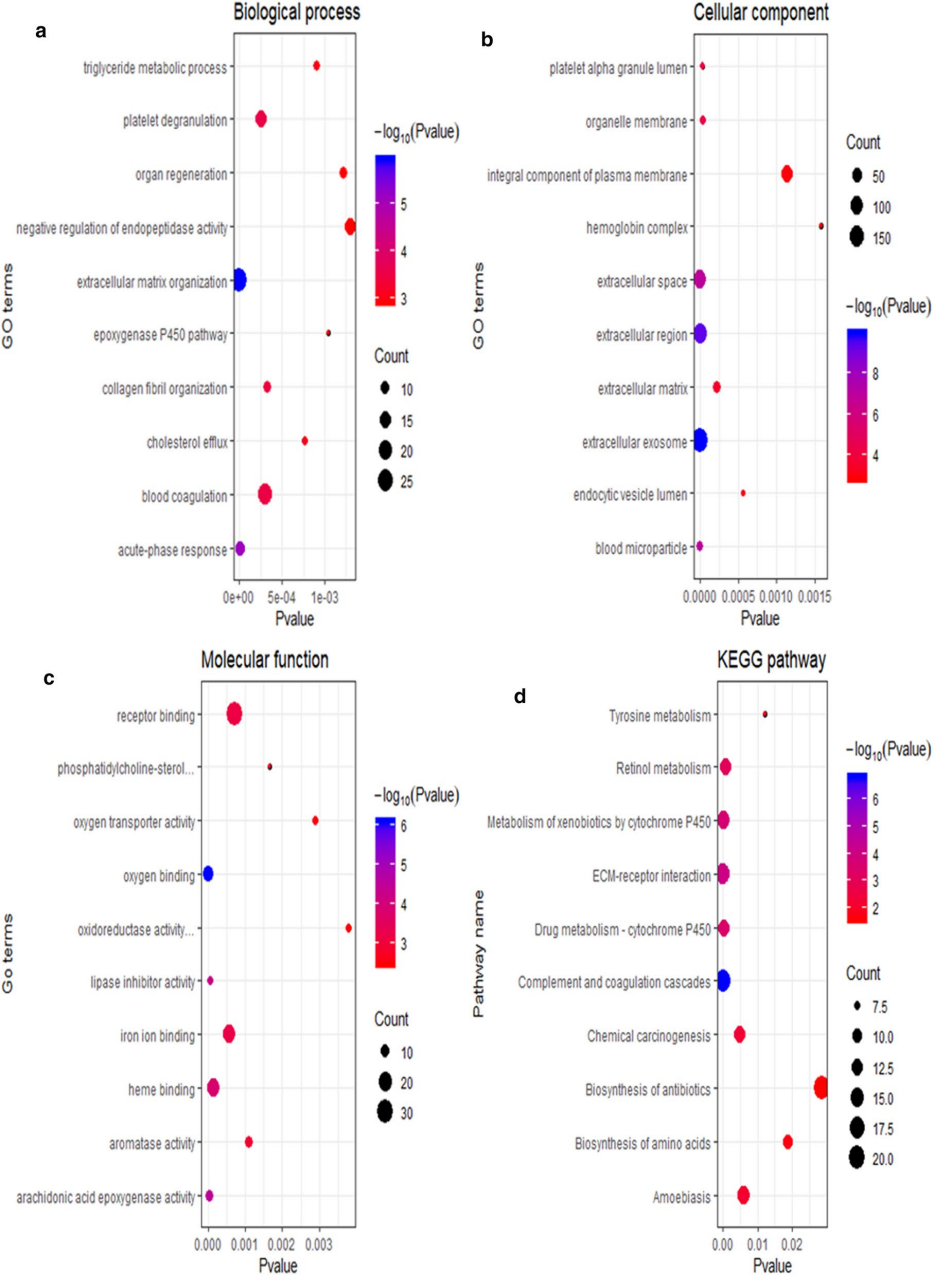
Functional enrichment analysis. **a** The top 10 enriched terms in biological process. **b** The top 10 enriched terms in cellular component. **c** The top 10 enriched terms in molecular function. **d** The top 10 enriched pathway in KEGG analysis

To further understand the potential biological functions of the identified DERNAs, we conducted GO and KEGG analyses using DAVID. The GO and KEGG analysis results with P < 0.05 were considered to be significant, and these results are displayed in Fig. 1. The significantly enriched GO terms in the biological process (BP) domain were extracellular matrix organization, acute-phase response, platelet degranulation, blood coagulation and collagen fibril organization (Fig. 1a). In the cellular component (CC) domain, the significantly enriched terms in which the DERNAs were involved were extracellular exosome, extracellular region, blood microparticle and platelet alpha granule lumen (Fig. 1b). In the molecular function (MF) domain, the DERNAs were mainly enriched in oxygen binding, arachidonic acid epoxygenase activity, lipase inhibitor activity, heme binding and iron ion binding (Fig. 1c). The KEGG pathway analysis revealed that the genes were mainly associated with Complement and coagulation cascades, ECM-receptor interaction, Metabolism of xenobiotics by cytochrome P450, Drug metabolism—cytochrome P450, Retinol metabolism and Chemical carcinogenesis (Fig. 1d).

### Survival analysis and Validation of DERNAs

**Fig. 2:**
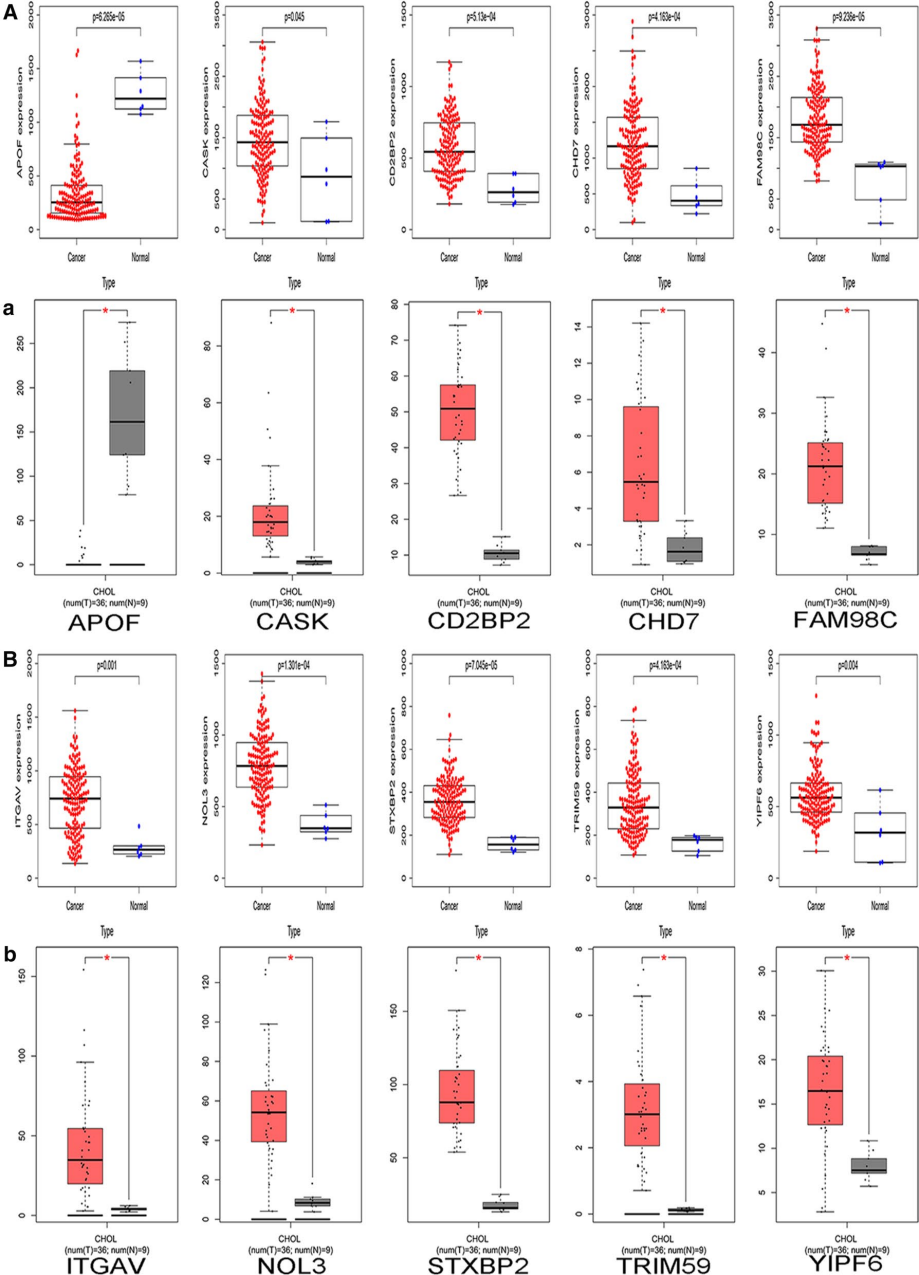
Validation of the DERNAs. **A**, **B** Scatter plots of expression of DERNAs associated with survival in the GSE32225 dataset. **a**, **b** The expression levels of DERNAs associated with survival in TCGA dataset. Red block is CCA tissues, * stands for P < 0.05, and the difference is statistically significant

**Fig. 3:**
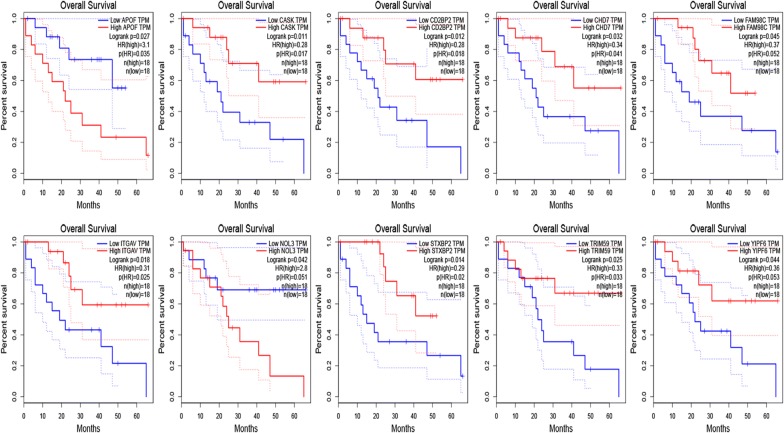
Survival analysis of the DERNAs. The relationship between DERNAs and survival of CCA patients was evaluated by performing Kaplan–Meier survival curves

By using TCGA data for survival analysis, we evaluated the prognostic value of these DERNAs. The GEPIA analysis tool is based on clinical data from the TCGA database, and clinical data from 36 patients were incorporated in the survival analysis. The results indicate that a total of 18 out of 875 DERNAs were observably associated with OS. Then, with the help of GEPIA, the expression of survival related DERNAs was verified in the TCGA data. We found that the CASK, APOF, NOL3, CD2BP2, CHD7, FAM98C, STXBP2, TRIM59, YIPF6 and ITGAV expression levels were consistent with our analysis results (Fig. 2). We then removed the inconsistent DERNAs in further analyses. The validated survival-related DERNAs are shown in Fig. 3.

### Differentially expressed proteins (DEPs) and integrated analysis

**Fig. 4:**
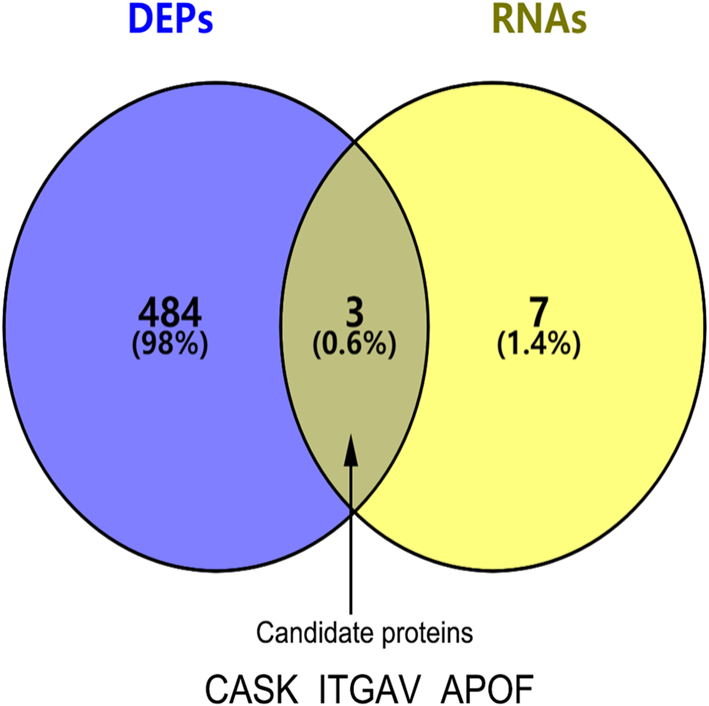
Integrated data analysis. Venn diagram present the intersection of DEPs with survival related DERNAs

By using the iTRAQ technique, we identified a total of 2886 confidential proteins in 6 matched primary and nontumour tissues. Among these, 487 proteins showed statistically significant in differential expression between CCA and paracancerous tissues, among which 235 was upregulated and 252 downregulated. We integrated the DEP data with the prognosis-related DERNA data validated by the TCGA dataset. By intersecting the two sets of data, we obtained 3 candidate proteins for further study (Fig. 4). Figure 4 was created by using the VENNY 2.1 tool (https://bioinfogp.cnb.csic.es/tools/venny/index.html). These candidate proteins are summarized in Table 3.

**Table 3:** The Basic Properties of candidate protein

Accession no.	Gene symbol	Sequence coverage (%)	Peptides	Gene ID
sp|O14936|CSKP_HUMAN	CASK	9.5	5	8573
sp|Q13790|APOF_HUMAN	APOF	24.7	16	319
sp|P06756|ITAV_HUMAN	ITGAV	4.3	1	3685

### ITGAV or APOF expression in choledochal cysts and CCA

**Fig. 5:**
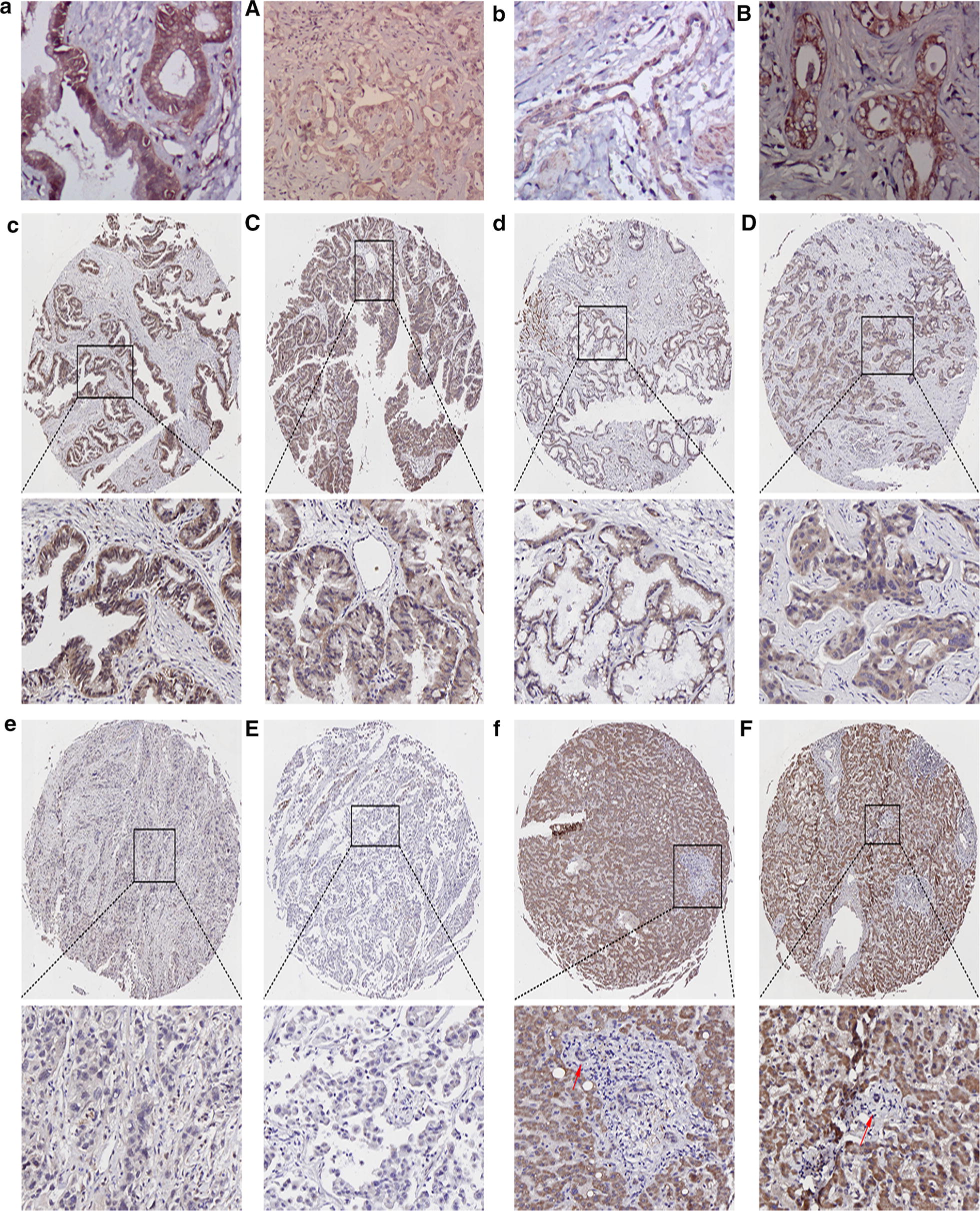
Immunohistochemical assay. **a** APOF is highly expressed in choledochal cyst. **A** APOF is lowly expressed in CCA. **b** ITGAV is poorly expressed in choledochal cyst. **B** ITGAV is moderately expressed in CCA. **c**, **C** CASK was strongly positive expressed in CCA.** d**,** D** CASK was moderately positive expressed in CCA. **e**, **E** CASK was weakly positive expressed in CCA.** f**,** F** CASK was completely negative expressed in interlobular bile duct. The red arrow shows the interlobular bile duct

APOF was highly expressed in the cytoplasm of 7 choledochal cysts (Fig. 5a), and ITGAV was poorly expressed in the cell cytoplasm of 2 choledochal cysts (Fig. 5b). APOF was expressed at low levels in the cytoplasm in 3 of 14 CCA cases (Fig. 5A). ITGAV was expressed in the cytoplasm in 6 of 14 CCA tissues (Fig. 5B).

### CASK expression in TMA

CASK expression was evaluated using a TMA comprising 31 interlobular bile ducts and 86 CCA tissues (Fig. 5). CASK was detected in the nuclei and cytoplasm of carcinoma cells in 38 of 86 CCA tissues in TMA. In the following analysis, 1 case was excluded due to incomplete follow-up data and 1 patient was excluded due to tissue loss.

### Relationship between CASK expression and clinicopathological features of CCA patients

The association between CASK expression and the clinicopathological characteristics of CCA patients is summarized in Table 4. CASK expression was found to be significantly correlated with vascular invasion (P = 0.009) and T classification (P = 0.017).

**Table 4:** The summary of relationship between CASK expression and clinicopathological features of CCA patients

Category	No. patients (%)	CASK		P value
		Negative	Positive	
Age				0.974
< 55	33 (39.3)	18	15	
≥ 55	51 (60.7)	28	23	
Gender				0.130
Female	43 (51.2)	27	16	
Male	41 (48.8)	19	22	
Histologic grade				0.300
Poorly	17 (20.2)	12	5	
Moderate	62 (73.8)	32	30	
Well	5 (6.0)	2	3	
Stage				0.491
I	8 (9.5)	3	5	
II	38 (45.2)	19	19	
III	25 (29.8)	16	9	
IV	13 (15.5)	8	5	
T classification				*0.017*
T1	6 (7.1)	2	4	
T2	58 (69.0)	30	28	
T3	17 (20.2)	14	3	
T4	3 (3.7)	0	3	
N classification				0.757
N0	56 (66.7)	30	26	
N1	28 (33.3)	16	12	
Vascular invasion				*0.009*
No	56 (66.7)	25	31	
Yes	28 (33.3)	21	7	
Recurrence				0.117
No	30 (35.7)	13	17	
Yes	54 (64.3)	33	21	

Italics values indicate P < 0.05

### Relationship between positive CASK expression and overall survival rate

**Fig. 6:**
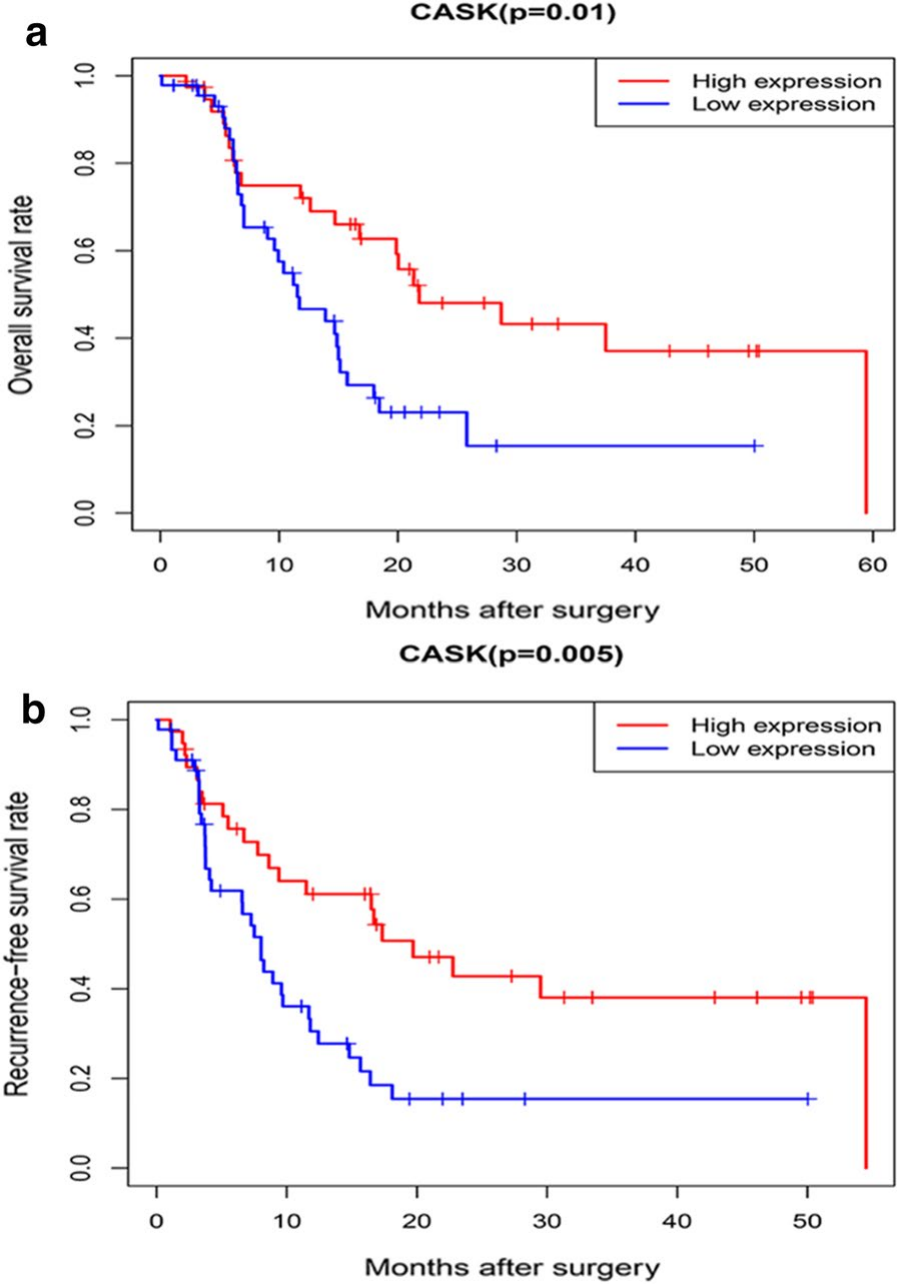
Kaplan–Meier survival analysis. **a** Kaplan–Meier survival analysis was used to analyse the relationship between CASK expression and OS of CCA patients. **b** Kaplan–Meier survival analysis was used to analyse the relationship between CASK expression and RFS of CCA patients

Kaplan–Meier curves showed that the survival rate of patients with positive CASK expression was significantly better than that of patients with negative CASK expression (P = 0.01; Fig. 6a). Univariate analysis of prognostic factors of CCA showed that pathological grade (P = 0.019), stage (P = 0.002), vascular invasion (P = 0.001) and negative CASK expression (P = 0.012) were significant risk factors for the outcome of CCA patients. Multivariate analysis showed that negative CASK expression (P = 0.027) was an independent risk factor for OS among CCA patients. The results of the univariate and multivariate analyses are summarized in Table 5.

**Table 5:** Univariate analysis and multivariate analysis of the relationship of CASK expression with OS among CCA patients

Parameter	Univariate analysis			Multivariate analysis		
	HR	95% CI	P	HR	95% CI	P
Age	1.018	0.560–1.854	0.952			
Gender	0.746	0.422–1.318	0.313			
Histologic grade	2.050	1.126–3.732	*0.019*	1.795	0.939–3.430	0.077
Stage	1.681	1.208–2.340	*0.002*	1.528	0.944–2.474	0.085
T classification	1.262	0.852–1.869	0.246			
N classification	1.659	0.930–2.960	0.087			
Vascular invasion	2.644	1.501–4.656	*0.001*	1.236	0.523–2.918	0.629
CASK	2.155	1.188–3.909	*0.012*	2.031	1.083–3.809	*0.027*

Italics values indicate P < 0.05

*HR* hazard ratio, *CI* confidence interval

### Relationship between positive CASK expression and recurrence-free survival rate

As shown by the Kaplan–Meier curves for RFS in Fig. 6b, patients with CCA who expressed CASK positively were associated with a better RFS (P = 0.005). In univariate analysis, pathological grade (P = 0.017), stage (P = 0.001), vascular invasion (P = 0.00007) and negative CASK expression (P = 0.006) were independent risk factors for RFS in patients with CCA. The multivariate analysis showed that pathological grade (P = 0.036), stage (P = 0.035) and negative CASK expression (P = 0.014) were statistically significant independent risk factors among CCA patients. A summary of the univariate and multivariate analyses is shown in Table 6.

**Table 6:** Univariate analysis and multivariate analysis of the relationship of CASK expression with RFS among CCA patients

Parameter	Univariate analysis			Multivariate analysis		
	HR	95% CI	P	HR	95% CI	P
Age	1.157	0.655–2.045	0.616			
Gender	0.835	0.485–1.437	0.515			
Histologic grade	1.983	1.130–3.481	*0.017*	1.918	1.044–3.526	*0.036*
Stage	1.750	1.273–2.408	*0.001*	1.612	1.034–2.514	*0.035*
T classification	1.377	0.946–2.004	0.095			
N classification	1.591	0.910–2.780	0.103			
Vascular invasion	2.797	1.625–4.816	*0.000*	1.325	0.616–2.851	0.472
CASK	2.229	1.259–3.947	*0.006*	2.114	1.163–3.844	*0.014*

Italics values indicate P < 0.05

*HR* hazard ratio, *CI* confidence interval

## Discussion

In most published studies, many single-molecule biomarkers have been identified and clinically applied for CCA; for example, CA19-9 can be used as a diagnostic marker for CCA [20], SMAD4 expression is associated with the prognosis of CCA patients [21], and PPP3CA expression is an independent prognostic risk factor for CCA patients [22]. With the continuous improvement of multiomics technology, we can now screen cancer biomarkers at different levels through the combined application of multiple omics data to avoid the randomness of single omics data, improve the accuracy of diagnostic and prognostic biomarkers, and search for possible therapeutic targets [23, 24]. However, the special clinical characteristics of CCA, combined with restrictions from limited, CCA-specific research funds, make it difficult to obtain CCA tissue samples and therefore to realize the combined application of multiple omics to analyse this data. Fortunately, as bioinformatics has continued developing and public databases such as GEO and TCGA have been established, the realization of combined multiomics applications has become more acceptable. By sharing transcriptome microarray data uploaded by experimenters around the world, we can apply combinations of multiple omics techniques to provide a comprehensive and systematic perspective to advance the understanding of the molecular mechanism of CCA [25–27].

In our study, we first obtained CCA transcriptome data from the GEO database by using a bioinformatics method and then obtained 875 DERNAs through differential analysis of the microarray data by R software language. We further performed GO and KEGG functional analysis on these DERNAs. These DERNAs were basically enriched in the pathways of Complement and coagulation cascades, ECM-receptor interaction, Metabolism of xenobiotics by cytochrome P450, Drug metabolism—cytochrome P450, Retinol metabolism and Chemical carcinogenesis, which are closely related to tumour occurrence and development. Then, we used GEPIA to analyse CCA transcriptome data and the corresponding clinical information in the TCGA database. We explored the relationship between these DERNAs and the prognosis of patients and verified the differential expression of these prognostic related DERNAs in the TCGA data. We found that CASK, APOF, NOL3, CD2BP2, CHD7, FAM98C, STXBP2, TRIM59, YIPF6 and ITGAV expression were associated with CCA prognosis. In addition to reports that TRIM59 can be knocked out to inhibit the proliferation of CCA [28], other DERNAs were reported for the first time to show differences between tumour and normal tissue and were significantly associated with the prognosis of CCA patients.

Subsequently, we used the iTRAQ quantitative proteomics technique to screen out DEPs between CCA and nontumour tissues. By using the iTRAQ technique, we found 487 DEPs, of which 235 were upregulated and 252 were downregulated. By integrating transcriptome data with proteome data, we found that CASK, ITGAV and APOF were differentially expressed at the mRNA and protein levels. Through survival analysis, these DERNAs can serve as prognostic biomarkers for CCA. Through multiomics joint analysis, it was found that the proteins that were translated from these DERNAs were also differentially expressed between CCA and nontumour tissue. Therefore, these candidate proteins are likely associated with the prognosis of patients and may help us to understand the occurrence and development of CCA from a systematic and comprehensive perspective. Given the above reasons and the results of the literature review, we believe that ITGAV, APOF and CASK may be potential protein prognostic biomarkers and thus conducted further research on them. We used immunohistochemistry to identify the expression profiles of these candidate proteins in CCA tissues. After a preliminary evaluation of the immunohistochemical results by the immunoreactivity score, we found that the differential expression of CASK between CCA and nontumour tissues was significant. We further evaluated CASK expression in 84 CCA patients and explored the association between CASK expression and prognosis in patients with CCA.

Integrin subunit alpha V (ITGAV), a member of the integrin family, has been found to be associated with cell differentiation and metastasis and can serve as a prognostic biomarker for a variety of cancers [29–31]. In our study, we found that ITGAV mRNA expression was significantly upregulated in CCA and that upregulated expression was associated with better prognosis in CCA patients through bioinformatics analysis. Through iTRAQ quantitative proteomics, we found that the expression of ITGAV protein in CCA samples was stronger than that in nontumour samples, which was consistent with the results at the mRNA level. This finding is also consistent with the expression of ITGAV in gastric cancer, but overexpression of ITGAV has been found to be associated with a poor prognosis in gastric cancer patients [29]. Kang et al. reported that lncRNA AY can promote hepatocellular carcinoma metastasis by inducing transcription of ITGAV [32]. ITGAV may promote or inhibit cancer according to the type of tumour [33, 34]. Therefore, further research is needed to clarify its mechanism in the development of carcinoma.

Apolipoprotein F (APOF) is a lipid transfer inhibitor protein that can inhibit the activity of cholesteryl ester transfer protein (CETP) and plays an important role in lipid metabolism [35–37]. Wang et al. found that the expression of APOF at both the mRNA and protein levels in hepatocellular carcinoma was significantly downregulated, and low APOF expression was associated with poor RFS in hepatocellular carcinoma patients [38]. In this study, we found that APOF expression at both the mRNA and protein levels in CCA was significantly downregulated compared with in noncancer tissues. Through survival analysis, we found that the OS of CCA patients with high APOF expression at the mRNA level was significantly lower than that of other CCA patients. The mechanism of APOF in CCA is unclear and may be related to lipid metabolism. Future research is needed to clarify the relationship between APOF in CCA and lipid metabolism, which may provide new ideas for research on CCA.

Peripheral plasma membrane protein CASK (CASK), also known as calcium/calmodulin-dependent serine protein kinase, is a member of the membrane-related guanylate kinase (MAGUK) family, a group of cytoskeletal proteins composed of modular domain arrays [39, 40]. CASK, which has been intensively studied in neurons, is deemed to be an important histone protein in cortical networks and may be involved in the establishment and maintenance of synaptic connections [41–44]. The role of CASK in carcinoma has been reported only in oesophageal cancer, gastric cancer and colorectal cancer [45, 46]. Wang et al. found that CASK expression in oesophageal cancer was upregulated at the mRNA and protein levels [39]. Wei et al. reported that CASK is highly expressed in colorectal cancer and is associated with poor prognosis in colorectal cancer patients [47]. In this study, we first found that CASK was upregulated at the mRNA and protein levels in CCA compared with nontumour tissue by using bioinformatics and proteomics. Through Kaplan–Meier survival analysis, we found that the upregulated CASK expression at both the mRNA and protein levels was significantly related to a better prognosis in CCA patients and that CCA patients with high CASK expression at the protein level had a longer recurrence-free survival rate, which means that low CASK expression is an independent prognostic risk factor in CCA patients. Moreover, univariate and multivariate analyses also showed that low CASK expression was an independent risk factor for recurrence and poor prognosis.

The mechanism by which CASK plays a role in the development of carcinoma is unclear. It has been reported that the interaction and coexpression of CASK and CX43 can affect cell migration [48]. Zhou et al. found that the expression of miR-203 can inhibit the growth and invasion of gastric cancer cells by inhibiting the expression of CASK, which may provide a new idea for explaining how CASK is involved in the development of tumours from the perspective of a competing endogenous RNA (ceRNA) network [46]. Interestingly, when we studied the TCGA database by using the GEPIA web tool, we also found that patients with high CASK expression tended to have poorer OS and RFS than other hepatocellular carcinoma patients. This also indicates that CASK may be located upstream of tumour-related pathways and may modulate the entire network in a complex manner, showing different roles depending on the type of tumour. Therefore, the potential role of CASK in the process of tumourigenesis or tumour development and its mechanism deserve further investigation.

Through bioinformatics prediction, we can more easily find and verify candidate proteins that may be used as prognostic markers of CCA. Moreover, the integrated analysis of multiomics data will help us to explore the mechanism of CCA from a holistic perspective. However, due to a limited funding situation, we were only able to evaluate CASK as a prognostic marker for CCA, and the mechanism of CASK’s role in CCA remains unclear. Further research on this issue will help to elucidate the molecular mechanism of the occurrence or progression of CCA and may provide potential therapeutic targets for the treatment of CCA.

## Conclusions

In summary, we used bioinformatics analysis to identify 875 DERNAs and explored their potential functions in relation to CCA. Ten prognostic related DERNAs that have not been previously reported were screened out. By using the iTRAQ technique, we identified 487 DEPs between CCA and nontumour tissues. Multiomics integrated analysis revealed differences at the mRNA and protein levels for CASK, ITGAV and APOF in CCA tissues compared to nontumour tissues. Low expression of CASK at the mRNA level is associated with a poor prognosis in CCA patients. Low CASK expression at the protein level is an independent risk factor for recurrence and poor prognosis in CCA patients. Our study found a reliable method for the screening of biomarkers for CCA and may provide a comprehensive and systematic perspective for the in-depth study of the pathogenesis of CCA.

## Supplementary information

**Additional file 1.** The data of differentially expressed RNAs in GSE32225.

**Additional file 2.** Volcano plots of the DERNAs.

**Additional file 3.** Heatmap of the DERNAs.

## Data Availability

The data used to sustain the results of this work are included in the article and its supplementary information files.

## Abbreviations

CCA: Cholangiocarcinoma
DERNAs: Differentially expressed RNAs
DEPs: Differentially expressed RNAs
GEO: Gene Expression Omnibus
TCGA: The Cancer Genome Atlas
GO: Gene Ontology
KEGG: Kyoto Encyclopedia of Genes and Genomes
iTRAQ: Isobaric tags for relative and absolute quantitation
CASK: Peripheral plasma membrane protein CASK
ITGAV: Integrin subunit alpha V
APOF: Apolipoprotein F
IHC: Immunohistochemistry
DAB: Diaminobenzidine
OS: Overall survival
RFS: Recurrence free survival
LC–MS: Liquid chromatography–mass spectrometry
TMA: Tissue microarray
SCX: Strong cation exchange
DAVID: Database for Annotation, Visualization, and Integrated Discovery
GEPIA: Gene Expression Profiling Interactive Analysis
FC: Fold change
