# Supplementary figures and images for: Bioinformatics combined with quantitative proteomics analyses and identification of potential biomarkers in cholangiocarcinoma
Source: Cancer Cell Int. 2020 Apr 22;20:130. doi: 10.1186/s12935-020-01212-z (PMC7178764; doi:10.1186/s12935-020-01212-z)

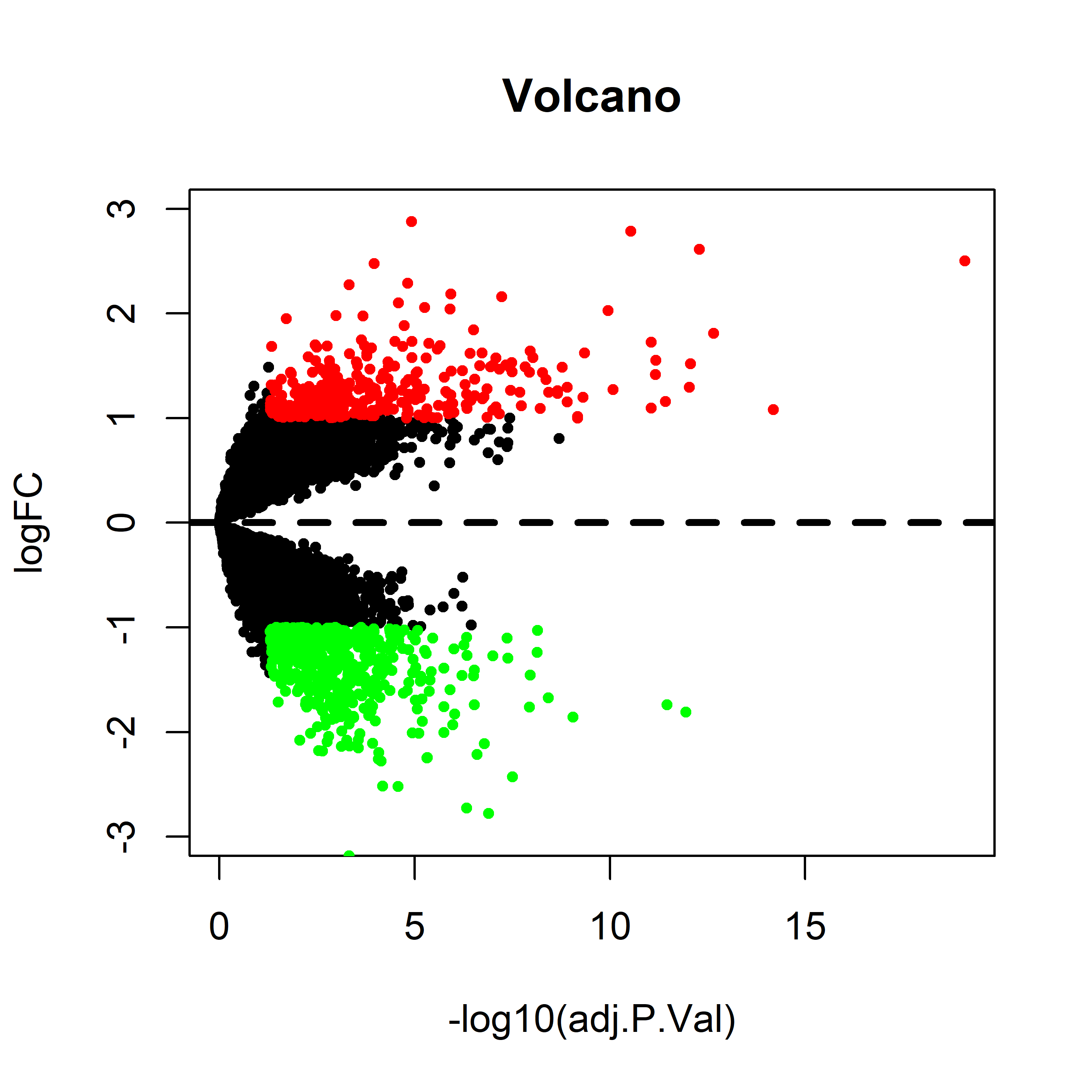

Supplement: Supplementary file 2 — Additional file 2. Volcano plots of the DERNAs. [file 12935_2020_1212_MOESM2_ESM.tiff]

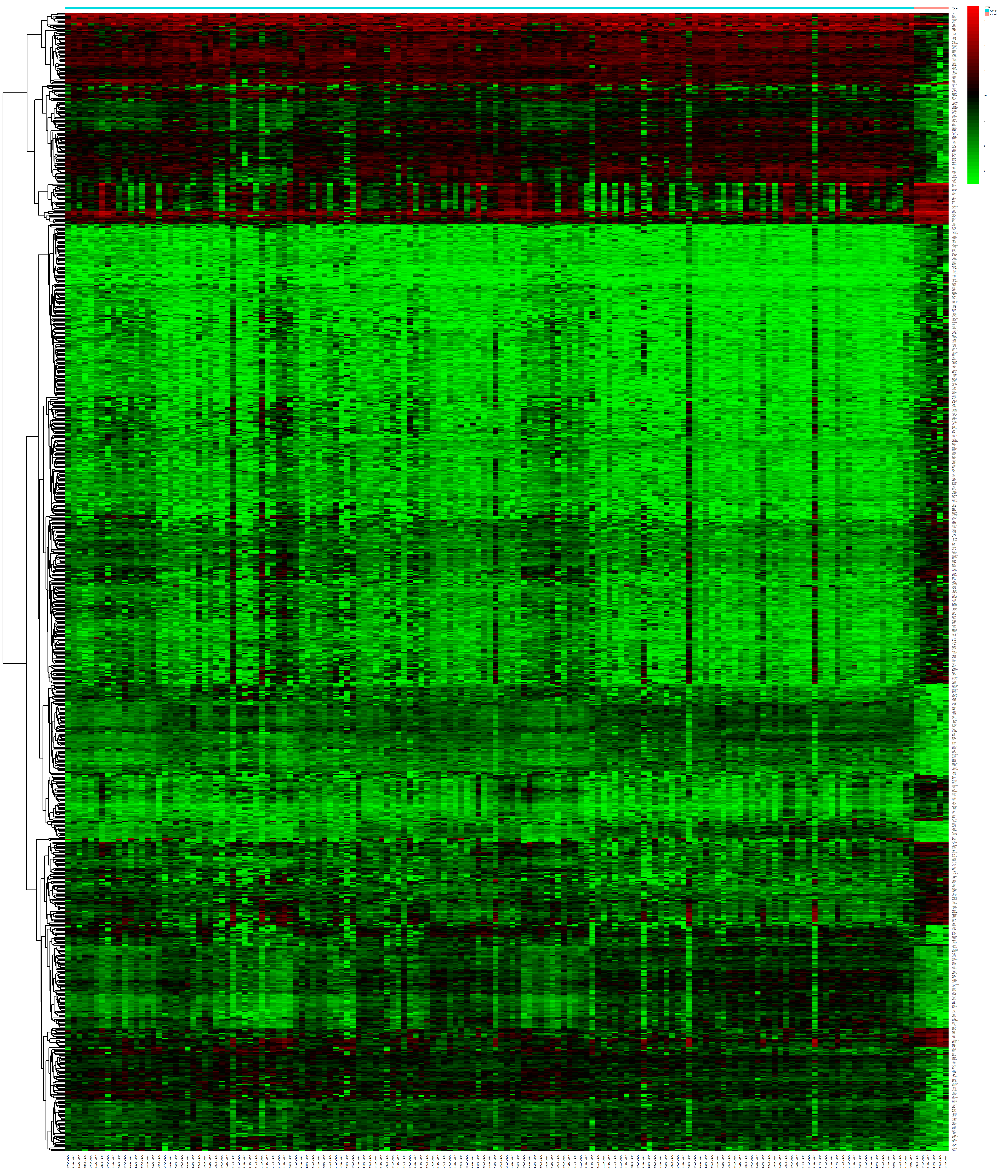

Supplement: Supplementary file 3 — Additional file 3. Heatmap of the DERNAs. [file 12935_2020_1212_MOESM3_ESM.tiff]
